# The Role of Early Engagement in a Self-Directed, Digital Mental Health Intervention for Adolescent Anxiety: Moderated Regression Analysis

**DOI:** 10.2196/60523

**Published:** 2025-06-02

**Authors:** Emma-Leigh Senyard, Arlen Rowe, Govind Krishnamoorthy, Susan H Spence, Caroline Donovan, Sonja March

**Affiliations:** 1 School of Psychology and Wellbeing, University of Southern Queensland, Ipswich, Australia; 2 Centre for Health Research, University of Southern Queensland, 37 Sinnathamby Boulevard, Springfield Central, 4300, Australia, 61 73812 6155; 3 Manna Institute, Springfield Central, Australia; 4 Australian Institute for Suicide Research and Prevention, Griffith University, Mount Gravatt, Australia; 5 School of Applied Psychology, Griffith University, Mount Gravatt, Australia; 6 Griffith Centre for Mental Health, Griffith University, Mount Gravatt, Australia

**Keywords:** youth, adolescent, teen, teenager, anxiety, eHealth, internet, engagement, mental health, participation, cognitive behavioral therapy, CBT, psychotherapy, self-help, self-guided, retention, attrition, dropout, digital mental health, web-based

## Abstract

**Background:**

Digital mental health (dMH) interventions offer the ability to reach many more adolescents with anxiety than face-to-face therapy. While efficacious dMH interventions are available for adolescents, premature dropout and low engagement are common, especially if delivered on a self-help basis without any form of therapist guidance. This is concerning, given that higher engagement, in terms of the number of sessions completed, has been repeatedly associated with improved clinical outcomes. The reasons for poor adolescent engagement in dMH programs are unclear. A clear understanding of when and why disengagement occurs is important in order to seek to improve engagement rates. Contemporary models consider engagement as multifaceted, comprising both “use” (eg, amount of content completed, frequency of use, duration spent logged into the dMH program, and depth of use, such as word or character count) and “user experience” (eg, interest and satisfaction in the program and affect and attention whilst engaging in the program).

**Objective:**

This study investigated the role of demographic and early engagement (EE) factors, specifically program use, in predicting overall program engagement and continued engagement, respectively, in a self-directed, internet-based cognitive behavioral therapy program for adolescent anxiety, namely, BRAVE Self-Help. It examined multiple measures of program use, including task completion, homework completion, and depth of response (character count of responses typed into program tasks). It also examined the moderating role of baseline anxiety severity.

**Methods:**

Data collected between July 2014 and May 2020 from 2850 adolescents aged 12 to 18 years who participated in BRAVE Self-Help were analyzed via a series of moderated regressions.

**Results:**

Results showed that EE (in terms of program use) was associated with continued engagement, demonstrated by early tasks (tasks completed in the first two sessions; *R*^2^=0.035; *P*<.001) and early depth (characters written in the first two sessions; *R*^2^=0.08; *P*<.001) predicting continued depth of program response (total character count of responses typed into all program tasks from sessions 3 to 10). Demographic factors and anxiety severity did not directly impact adolescents’ engagement in BRAVE Self-Help.

**Conclusions:**

These findings highlight the need to investigate ways to (1) enhance EE and (2) better understand how to measure and capture all aspects of program engagement.

## Introduction

### Background

Approximately 6.5% of adolescents internationally experience anxiety [1]. Anxiety can become entrenched, lead to a lifelong struggle, and is linked to numerous other problems, such as reduced academic performance, poor self-esteem, social problems, substance abuse, depression, and attention and concentration difficulties [2,3]. Digital mental health (dMH) interventions are an effective treatment of choice for many adolescents with anxiety [4-6]. These digital interventions are highly accessible, flexible, affordable, require no waitlists, and reduce concerns about stigma [6].

While dMH interventions are highly accessible and efficacious, it is a concern that, when delivered on a self-directed basis without therapist support, many adolescents cease the program early or neglect to complete program activities [4]. For example, in a study on the dMH program, youthCOACH, which targets chronic medical conditions in youth aged 12 and 21 years using cognitive behavioral therapy (CBT), only 40% of adolescents adhered to the intervention (completed at least 80% of the seven modules) [7]. In another study on the BRAVE Self-Help program, an open-access CBT dMH program for child and adolescent anxiety, only 30% of the 4425 children and adolescents completed more than three sessions, with the greatest clinical improvements made by those users completing at least 6 of 10 sessions [4]. These findings are consistent with those of a recent systematic review and meta-analysis of 35 predominantly adult dMH interventions (both therapist-supported and self-help), which found that greater engagement was associated with stronger treatment gains [8]. Thus, those with lower engagement potentially fail to obtain the greatest clinical benefit.

### Engagement in dMH Interventions

Gan et al [8] suggested that variability in the operationalization of engagement is a limitation of the dMH field, and defined engagement as any objective indicator of intervention use (eg, sessions completed, time spent, or number of logins). Indeed, a broader inspection of the literature reveals that dMH researchers have not applied a universal definition of the construct of engagement, and the term is frequently used interchangeably with “adherence,” “compliance,” and “use” [9,10]. To date, the majority of research has focused on the amount of the intervention completed, such as the number of completed treatment sessions or activities (eg, March et al [4], Calear et al [11], and Fleming et al [12]) to capture the use of dMH interventions. However, it is now widely acknowledged that engagement is multifaceted and incorporates more than just the number of sessions completed [4,13,14]. Two key reviews by Yardley et al [14] and Perski et al [15] suggest that engagement should be conceptualized as including both use and user experience. Use refers to the amount of intervention content completed (ie, completed sessions and activities), frequency of session completion, duration spent on intervention, and depth of responses (ie, character or word count). In contrast, user experience refers to subjective, self-reported experiences (ie, attention, interest, and satisfaction with the program), and affect experienced during the program [14,15]. The clinical validity and reliability of these constructs are not yet known; however, the frameworks proposed by Yardley et al [14] and Perski et al [15] provide a useful and comprehensive structure to guide research and advance our understanding and measurement of the numerous potential facets of engagement in dMH interventions.

### Early Engagement

Given that higher program use (at least in terms of the number of sessions completed) has consistently been shown to be positively associated with treatment outcomes, but that many participants do not progress beyond a few sessions of dMH programs [8,16], it appears that understanding early engagement (EE) in the first few sessions could offer valuable insight and assist in identifying ways to enhance overall program completion. In a blended-care CBT intervention (combination of videoconference therapy sessions and personalized dMH activities), Wu et al [17] examined the predictors of program retention and found that engagement was poor if participants did not complete their assigned web-based activities early in treatment. Here, engagement was defined as the completion of both the first telehealth therapy session and assigned dMH activities after the session. In relation to self-help dMH programs, there is some research that has identified early “use” in dMH programs is related to program engagement, however, evidence is limited to engagement conceptualized as word count of activity responses [18]. For example, Wallert et al [18] found that writing more words in early dMH activities was related to higher rates of overall program adherence (completing three or more homework tasks). It is possible that greater effort or more in-depth use of content and tasks early in the program (better engagement) may facilitate interest, trigger symptom improvements, and sustained engagement over time. Given that more recent frameworks propose the conceptualization of engagement beyond the number of program sessions completed, there is a need to examine additional facets of engagement and determine whether different types of EE (eg, early completion of in-session and homework tasks, character count, subjective self-report measures, and affect experienced during the program) are important in facilitating program engagement in dMH interventions. This study focuses specifically on the program use component of engagement as it is typically routinely collected and operationalizes multiple measures of program use. It also focuses primarily on the role of EE as described above, and its relationship to overall engagement.

### Factors Influencing dMH Engagement

Much of the existing literature has examined factors associated with dMH engagement in adult populations. In a review of 83 adult dMH programs, Liverpool et al [19] found that both intervention-specific influences (such as program suitability, usability, and acceptability) and person-specific influences (such as motivation, opportunity and time availability, family support, and capability) influenced user engagement. Borghouts et al [20] further highlighted that social connectedness, participants feeling “in control” of their health, and having insight into their concerns, were also facilitators of dMH program engagement. With respect to adolescent programs, a review of dMH programs by Lehtimaki et al [21] found that female, those with lower baseline anxiety, and those with a longer history of mental health conditions were more likely to complete dMH interventions [21]. Similarly, March et al [4,16] found that male, those with higher baseline anxiety severity, and those living in nonmetropolitan regions showed lower levels of engagement (operationalized as the number of sessions completed) in a dMH program for anxiety. However, to date, there is very limited research specifically examining the individual facets of engagement (as suggested in the frameworks by Perski et al [15] and Yardley et al [14]) that predict program engagement with dMH interventions by adolescents.

### Aims of This Research

This study examined the factors influencing program engagement, specifically, program use, in a dMH intervention for adolescent anxiety (BRAVE Self-Help). Extending on previous literature, engagement was conceptualized as being multifaceted. The first aim was to examine the effect of person-specific demographic factors (including gender, age, and residential location) on program engagement (sessions completed, in-session and homework tasks completed, depth (total character count), and average frequency (days) between sessions). The second aim was to examine the impact of EE on continued program engagement. EE was examined specifically in relation to program use, and conceptualized as the number of in-session tasks completed, homework tasks completed, and character count (depth of use), within the first two sessions of the program. In addition, previous research has found that participants completing a greater number of BRAVE sessions showed lower baseline anxiety severity [4] and were less likely to be classed in the high anxiety severity group when examining trajectories of change [16], suggesting that anxiety severity may be a relevant factor to consider. Therefore, this study further examined whether baseline anxiety severity moderated the relationship between demographic and EE factors and program engagement.

## Methods

### Participants and Procedure

Participants were 2850 adolescents aged between 12 and 17 years who had registered for the BRAVE Self-Help program between July 2014 and May 2020. In line with the intended audience of BRAVE Self-Help, only participants demonstrating baseline anxiety severity at an elevated or clinical level on the Children’s Anxiety Scale 8-item (CAS-8; see below for details) [22] were included in this study. This study was restricted to adolescents who had completed at least 2 sessions of the program. This was in line with our conceptualization of EE in this study. To ensure adequate measurement of EE, data were required relating to in-session tasks completed (sessions 1 and 2), and between-session tasks (homework allocated in session 1 that was subsequently reported on in session 2). Thus, to provide a comprehensive indication of EE, we required data collected in sessions 1 and 2. Further, as per calls for research to more comprehensively examine engagement through multiple components (eg, Yardley et al [14] and Perski et al [15]), it was important to include 2 sessions in our definition of EE as many dMH programs focus only on introductory and education-based content in the first session (eg, March et al [16]) without the inclusion of many interactive activities. Therefore, examining only responses to session 1 would not allow sufficient examination of program use in the form of completed activities or use depth. Participants were excluded from the study if they were completing the program as part of a school curriculum or school-based intervention, in order to mitigate any other engagement strategies delivered to these participants (eg, mandatory attendance and homework completion and support or facilitation by a school professional).

### Ethical Considerations

Participants were self-referred or referred by others (eg, general practitioners, school counselors, and other mental health practitioners) to the open-access BRAVE Self-Help program. No incentives were provided for participating in the program. Participants provided informed consent prior to commencing the program, and parental consent was also obtained if the adolescent was younger than 16 years of age. Informed consent was also provided for their data to be used in future research. All data analyzed were deidentified. During the registration process, participants provided demographic information (see below) and completed the CAS-8 [22]. This study was part of a larger, ongoing effectiveness study of BRAVE Self-Help (human research ethics approval: ETH2023-0832; University of Southern Queensland; 2020/581; Griffith University).

### Intervention

This study used data extracted from BRAVE Self-Help for teenagers (aged 12 to 17 years), a dMH intervention for adolescents with anxiety [4]. BRAVE Self-Help is delivered in 10 web-based, interactive, weekly sessions of 30 to 60 minutes duration and includes cognitive-behavioral strategies such as psychoeducation, cognitive restructuring, physiological awareness, relaxation, graded exposure, problem-solving strategies, positive reinforcement, and guided imagery [23]. Homework tasks are allocated each week to consolidate and enhance learning from each session. Reminders to complete sessions are sent to participants via automated email. BRAVE Self-Help is completed without therapist support, and sessions can be completed at any time but must be completed in order. For a detailed description of the program, refer to March et al [4].

### Measures

#### Demographics

Demographic information was measured at program registration and included age (years), gender, and location (postcode). Participants selected their gender from male, female, transgender, transsexual, transgender or transsexual, genderqueer, androgynous, or other. For analytic purposes, gender was recoded into male, female, and other. Those who identified their gender as “other” were excluded from analyses due to the small number of participants in this category (n=3). Location was coded according to the Australian Statistical Geography Standard [24] with participants categorized into: Major Cities, Inner Regional, Outer Regional, Remote, and Very Remote locations. For analytic purposes and due to the small number of participants in Outer Regional, Remote, and Very Remote categories, the location was recoded into three categories: (1) Major Cities, (2) Inner Regional, and (3) Outer Regional and Remote.

#### Anxiety Severity

Anxiety symptom severity was measured at program registration through CAS-8 [22]. Respondents are required to rate on a 4-point scale from 0=Never to 3=Always, the frequency with which each item applies to them. Item responses are summed to produce a total anxiety symptom severity score between 0 and 24, with higher scores indicating greater anxiety symptom severity. CAS-8 scores above the 84th percentile (≥10 for male and ≥12 for female) were categorized as elevated, whilst scores above the 94th percentile (≥13 for male and ≥16 for female) were categorized as Clinical [22]. The CAS-8 has been shown to demonstrate high internal consistency (Cronbach α=0.89) [22]. The internal consistency of the CAS-8 in this study was good (Cronbach α=0.87).

#### Engagement

This study focused on engagement in terms of program use. A summary of all engagement measures and their operationalizations is provided in Table 1.

**Table 1.** Operationalizations of EE^a^ and OE^b^.

Variable	Definition	Method of measurement
EE		
Completed tasks in sessions 1 and 2 (early tasks)	Completed tasks were defined consistent with completed tasks for program engagement.	The total of the number of tasks completed in sessions 1 and 2, operationalized consistent with the program engagement definition for completed tasks, but for sessions 1 and 2 only.
Session 1 homework tasks completed (early homework)	Completed homework tasks were defined consistent with completed homework tasks for program engagement.	The number of session 1 homework tasks completed was measured, consistent with the definition for program engagement for homework tasks, but for sessions 1 and 2 only.
Depth of early responses (early depth)	Early depth was defined as the number of characters typed into free-text boxes in response to program activities.	The total number of characters included in free-text response boxes in sessions 1 and 2 was calculated. Homework tasks were not included.
OE		
Number of completed sessions (completed sessions)^c^	Adherence to prescribed program sessions.	The number of sessions completed.
Tasks completed (total tasks)^c^	Tasks were defined as an in-session activity or task embedded within the program, which required the participant to type in a textbox or respond to a question asked.	Total number of tasks completed throughout program. Each separate textbox or response activity was counted as one activity. Activities were defined as completed if the written response by the participant had a minimum of three characters. This was to reduce the probability of a participant filling in the textbox but not having engaged in the activity (ie, responding with an “x” or writing short answers). Each individual completed task was then computed to obtain the total number of tasks completed.
Overall homework tasks completed (total homework)^c^	Homework task completion was defined as a task embedded in the homework section of the session (as the start of the session), which required the participant to type in a textbox or respond to a question asked. Homework tasks were prescribed in the previous session and reported on in the following session.	The total number of homework tasks completed. Each separate textbox or response activity was counted as one activity. As with tasks completed, activities were defined as completed if the written response by the participant had a minimum of three characters.
Session frequency (frequency)^c^	Frequency was defined as how often a participant engages with the intervention.	The average number of days between each completed session.
Depth of responses (program depth)^c^	Program depth was defined as character count within program activities that required a written response. That is, the number of characters typed into free-text boxes.	Total number of characters typed into program activities. Homework tasks were not included. Character count was chosen (over word count) to minimize issues such as typing errors, and missing or additional spaces [25]. To compute the depth of responses, the total characters for each session was computed before averaging the total number of characters across completed sessions.

a EE: early engagement.

b OE: overall program engagement.

c These variables were measured across 10 sessions when demographic variables were the predictor of OE, but across sessions 3 to 10 when EE variables were the predictor of continued engagement.

### Early Engagement

EE was defined as engagement within the first 2 sessions of the program. Similar to the operationalization of engagement by Wu et al [17] , EE in this study was defined as the number of tasks completed in sessions 1 and 2, the number of session 1 homework tasks completed (data collected in session 2), and depth of responses (character count) in sessions 1 and 2. Because homework tasks allocated in session 1 are completed between sessions and reported in session 2, it was essential that this study included session 2 in its conceptualization of EE.

### Program Engagement

Program engagement was operationalized as both overall program engagement (OE) and continued engagement (CE). OE was measured through the total number of program sessions completed, total number of program tasks completed, total number of homework tasks completed, total engagement depth (character count of responses typed into any program tasks), and average frequency of sessions (average days between sessions). CE was defined as engagement after session 2 (ie, the number of in-session and homework tasks, sessions completed, and depth of engagement from sessions 3 to 10 only). The number of prescribed tasks and homework tasks per session is detailed in Multimedia Appendix 1.

### Analytic Strategy

Data were analyzed using SPSS Statistics (version 27; IBM Corp). EE and OE were computed from raw program output data and are described in Table 1 and reported in Table 2 for the total sample, elevated, and clinical subgroups. Differences between the elevated and clinical groups for overall and early program engagement were analyzed using Cohen *d*.

**Table 2.** Summary of participant demographic and clinical factors across baseline anxiety status.

Variable	Elevated anxiety(n=915, 32.11%)	Clinical anxiety(n=1935, 67.89%)	Total participants(n=2850)	*t* test (*df*)^a^	Cohen *d*^a^	Chi-square (*df*)^a^	*P* value^a^
Demographic factors							
Age (years), mean (SD)	13.84 (1.99)	14.32 (1.76)	14.17 (1.85)	6.513 (2848)	0.26	—^b^	<.001
Sex, n (%)				—	—	1.26 (1)	.26
Male	243 (26.55)	553 (27.55)	796 (27.92)				
Female	672 (73.44)	1382 (71.42)	2054 (72.07)				
Location, n (%)				—	—	0.73 (1)	.87
Major Cities	473 (51.69)	976 (50.44)	1449 (57.54)				
Inner Regional	214 (23.39)	471 (24.34)	685 (24.03)				
Outer Regional and Remote	119 (13.01)	265 (13.70)	384 (13.47)				
Missing^c^	109 (11.91)	223 (11.52)	332 (11.65)				
Aboriginal and Torres Strait Islander, n (%)				—	—	57.29 (1)	<.001
Yes	4 (0.01)	10 (0.01)	14 (0.01)				
No	199 (21.75)	214 (11.06)	413 (14.49)				
Missing^d^	712 (77.81)	1711 (88.42)	2423 (85.02)				
Clinical factors							
CAS-8^e^ score at baseline, mean (SD)	14.26 (4.41)	15.96 (6.54)	15.41 (5.99)	7.133 (2848)	0.30	—	<.001

a Ratio of difference, effect size (Cohen *d* and chi-square), degrees of freedom, and significance between the elevated and clinical group at baseline.

b Not applicable.

c 332 participants did not disclose their residing location.

d Aboriginal and Torres Strait Islander data were not collected until August 27, 2018.

e Children’s Anxiety Scale 8-item.

For the first research question, 15 separate moderated regression analyses were conducted to examine whether baseline demographic factors predicted OE (one moderated regression per predictor and dependent variable) and whether baseline anxiety severity moderated these relationships. Demographic factors (age, gender, and location) acted as the independent variables, whilst total completed sessions, total tasks completed, total homework tasks completed, depth of responses, and frequency of session completion acted as the (OE) dependent variables.

For the second research question, 15 separate moderated regression analyses were conducted to examine whether EE factors predicted CE (one moderated regression per predictor and dependent variable), and whether baseline anxiety severity moderated these relationships. Early tasks, early depth, and early homework acted as the independent variables. The dependent CE variables were completed sessions, total tasks completed, total homework tasks completed, depth of responses, and frequency of session completion.

Anxiety severity acted as the moderator variable, with participants categorized as either elevated or clinical. Regression analyses were conducted separately for each predictor, given that previous research has shown certain demographic factors may contribute more to overall engagement than others [4,21] and models suggest that the multifaceted nature of engagement needs to be further understood [14,15]. As we were interested in understanding which EE factors had the strongest effects in predicting CE, we conducted separate rather than multivariate regression analyses. Given the large number of regression analyses conducted, a conservative Bonferroni correction of *P*<.001 was applied for all analyses.

## Results

### Demographic and Clinical Characteristics of Participants

A summary of participant baseline demographic and clinical characteristics across anxiety status is provided in Table 2.

### EE, CE, and OE Characteristics

As seen in Table 3, adolescents in the elevated group demonstrated significantly greater OE than those in the clinical group, as measured by total homework, program depth, and early depth. Adolescents in the elevated group also had a significantly higher frequency (days between sessions) than those in the clinical group, which may suggest that these adolescents may be completing the program more in line with program frequency recommendations (sessions should be 5 to 7 days apart).

**Table 3.** Summary of OE^a^, CE^b^, and EE^c^ characteristics.

	Elevated group(n=915), mean (SD)	Clinical group(n=1935), mean (SD)	Total participants(n=2850), mean (SD)	Cohen *d^d^*	*P* value^d^
OE^e^					
Completed sessions (OE)	4.14 (2.60)	3.96 (2.52)	4.02 (2.55)	0.07	.08
Total tasks (OE)^f^	71.48 (40.67)	68.40 (39.66)	69.39 (40.01)	0.08	.06
Total homework^f^ (OE)	51.31 (30.93)	48.86 (29.85)	49.64 (30.22)	0.08	.04
Program depth (OE)	73.43 (52.10)	68.17 (49.90)	69.86 (50.67)	0.10	.01
Frequency (OE)	5.62 (8.25)	4.66 (8.31)	4.97 (8.30)	0.12	.004
CE^g^					
Completed sessions (CE)	5.17 (2.36)	5.11 (2.29)	5.13 (2.32)	0.03	.52
Total tasks (CE)^f^	119.07 (24.48)	116.77 (24.84)	117.63 (24.66)	0.09	.02
Total homework (CE)^f^	86.68 (24.59)	82.60 (25.77)	84.15 (25.33)	0.16	<.001
Program depth (CE)	767.10 (862.73)	673.67 (795.40)	703.67 (818.63)	0.11	.004
Frequency (CE)	6.43 (9.70)	4.82 (8.81)	5.33 (9.12)	0.17	<.001
EE^h^					
Early tasks (EE)^f^	16.97 (3.51)	16.98 (3.73)	16.98 (3.66)	0.00	.95
Early homework (EE)^f^	2.00 (2.00)	2.08 (2.00)	2.06 (2.00)	0.04	.32
Early depth (EE)	13.10 (7.07)	12.54 (6.94)	12.72 (6.99)	0.08	.05

a OE: overall program engagement.

b CE: continued engagement.

c EE: early engagement.

d Mean (SD), effect size, degrees of freedom, and significance between the elevated and clinical group at baseline.

e OE statistics across all 10 sessions of the program.

f The number of prescribed tasks and homework tasks per session is detailed in Multimedia Appendix 1.

g CE statistics (3 to 10 sessions only).

h EE statistics (session 1 and 2 only).

### Correlations Among Demographic Factors, OE, CE, and EE

Before completing moderated regressions, correlations were analyzed between all predictor and outcome variables, which are presented in Multimedia Appendix 2. Of the relationships of interest, greater age was significantly correlated with higher completed sessions, frequency, total homework, total tasks, and severity. Female gender was strongly associated with greater completed sessions and program depth. In relation to EE predictors, there was a significant correlation between higher early tasks and greater program depth, total homework, frequency, and total tasks. Greater early depth was also significantly positively associated with total homework, completed sessions, total tasks, and average frequency. Higher early homework was significantly and positively associated with program depth, total homework, total tasks, and frequency. Finally, lower anxiety severity was significantly correlated with greater program depth (CE), average frequency (CE), and program depth (OE).

### Demographic Factors Associated With OE

#### Overview

The results of the moderated regression analyses examining whether demographic variables predicted OE, and if these relationships were moderated by anxiety severity, are presented in Tables S1-S5 in Multimedia Appendix 3.

#### Completed Sessions (OE)

The overall regression models were significant for gender (*R*^2^=0.006; *P*<.001), but not for age (*R*^2^=0.004; *P*=.01), and location (*R*^2^=0.010; *P*=.01). Despite the overall model being significant, as shown in Table S1 in Multimedia Appendix 3, there were no significant unique effects for gender or interaction effects in predicting completed sessions (overall).

#### Total Tasks (OE)

The overall regression models were significant for age (*R*^2^=0.007; *P*<.001), but not for gender (*R*^2^=0.002; *P=.12*) or location (*R*^2^=0.007; *P*=.05). There were no significant unique effects for age or interaction effects (Table S2 in Multimedia Appendix 3).

#### Program Depth (OE)

The overall regression models for age (*R*^2^=0.003; *P*=.20), gender (*R*^2^=0.000; *P*=.60), and location (*R*^2^=0.004; *P*=.32) were not significant in predicting program depth (overall), nor were there any significant interactions (Table S3 in Multimedia Appendix 3).

#### Frequency (OE)

The overall regression models were significant for age (*R*^2^=0.079; *P<*.001), but not for gender (*R*^2^=0.003; *P=*.02) or location (*R*^2^=0.005; *P*=.02). There was no significant unique effect for age and no significant interaction effects observed (Table S4 in Multimedia Appendix 3). Whilst not significant at the Bonferroni level, there was a trend observed for the effect of severity in the regression model testing gender as the independent variable. There was a negative relationship between severity and frequency (overall), *r*_2188_=–0.082; *P*<.001, with higher levels of anxiety related to fewer days between sessions.

#### Total Homework (OE)

The overall models were not significant for age (*R*^2^=0.004; *P*=.11), gender (*R*^2^=0.002; *P*=.15), or location (*R*^2^=0.004; *P*=.23), nor were there any significant interaction effects (Table S5 in Multimedia Appendix 3).

### EE Factors Associated With CE

Outcomes from moderated regressions were conducted to determine whether EE variables predicted CE, and if these relationships were moderated by anxiety severity.

#### Completed Sessions (CE)

The overall regression models were significant for early depth (*R*^2^=0.011; *P*<.001), but not for early tasks (*R*^2^=0.004; *P*=.10) or early homework (*R*^2^=0.004; *P*=.09). Despite the overall model being significant (Table S1 in Multimedia Appendix 4), there were no unique effects for early depth and no significant interaction effects across any of the regression models.

#### Total Tasks (CE)

The overall regression models were significant for early homework (*R*^2^=0.236; *P*<.001), early tasks (*R*^2^=0.310; *P*<.001), and early depth (*R*^2^=0.166; *P*<.001) in predicting total tasks (CE). However, there were no significant unique effects or interactions at the conservative Bonferroni level, as shown in Table 4, though there were effects evident using a traditional *P*<.05 cut-off. Specifically, a trend was evident (*P*<.05) for a positive relationship between early homework and CE total tasks (*r*_2850_=0.481; *P*<.001), with greater homework tasks completed in sessions 1 and 2 related to greater overall task completion. This was also the case for early tasks and CE total tasks (*r*_2850_=0.555; *P*<.001), where greater tasks in the first 2 sessions were associated with greater CE tasks completed and between early depth and CE total tasks (*r*_2850_=0.405; *P*<.001), with more characters written in the first two sessions being associated with more total CE tasks completed.

**Table 4.** Linear model of EE^a^ variables predicting program tasks (CE^b^) as a function of anxiety severity.

EE predictors		β	Standard β coefficient	Individual regression coefficient (t)	*P* value	95% CI
Early tasks						
Constant		53.68	25.40	2.11	.04	3.57 to 103.80
Early tasks		1.77	0.70	2.53	.01	0.39 to 3.15
Severity		6.19	14.07	.44	.66	–21.56 to 33.94
Early tasks severity^c^		–.12	0.39	–0.30	.78	–0.89 to 0.66
Early homework						
Constant		55.15	24.03	2.30	.02	7.74 to 102.55
Early homework		2.73	1.03	2.65	.01	0.70 to 4.76
Severity		13.46	13.12	1.03	.31	–12.44 to 39.35
Early homework severity^c^		–.52	.57	–0.92	.36	–1.65 to 0.60
Early depth						
Constant		96.20	12.18	7.90	<.001	72.16 to 120.23
Early depth		.05	0.02	2.23	.03	0.01 to 0.09
Severity		2.50	7.04	0.37	.72	–11.40 to 16.40
Early depth severity^c^		–.01	0.01	–0.60	.56	–0.04 to 02

a EE: early engagement.

b CE: continued engagement.

c Severity refers to baseline anxiety severity.

#### Program Depth (CE)

The overall regression models were significant for early tasks (*R*^2^=0.035; *P*<.001), early homework (*R*^2^=0.03; *P*<.001), and early depth (*R*^2^=0.08; *P*<.001) in predicting program depth CE. As shown in Table 5, there was a significant unique positive effect for early tasks and early depth. CE program depth increased by 24.84 characters with each early task completed, explaining 3.5% of the variance in CE program depth. CE program depth also increased by 0.85 characters for each early depth increase of one character, accounting for 7.9% of the variance in CE program depth. Whilst not significant at the conservative Bonferroni significance level, there was a positive trend (*P*<.05) evident between early homework and CE program depth, *r*_2850_=0.154; *P*<.001, with CE program depth increasing as early homework tasks increased. No significant interactions were found in any of the regression models predicting CE program depth.

**Table 5.** Linear model of EE^a^ variables predicting program depth (CE^b^) as a function of anxiety severity.

EE predictors	β	Standard β coefficient	Individual regression coefficient (t)	*P* value	95% CI
Early tasks					
Constant	14.11	276.45	0.05	.96	–527.96 to 556.17
Early tasks	24.94	7.97	3.13	<.001	9.31 to 40.57
Severity	6.08	156.73	0.04	.97	–301.24 to 313.39
Early tasks severity^c^	–2.94	4.52	–0.65	.52	–11.80 to 5.92
Early homework					
Constant	264.36	216.70	1.22	.22	–160.53 to 689.25
Early homework	27.52	9.62	2.86	<.001	8.65 to 46.30
Severity	–11.54	123.25	–0.09	.93	–253.22 to 230.14
Early homework severity^c^	–3.85	5.47	–0.70	.48	–14.57 to 6.87
Early depth					
Constant	356.40	111.55	3.20	<.001	10.80 to 12.62
Early depth	.85	0.17	5.00	<.001	0.52 to 1.18
Severity	–28	63.80	–0.44	.66	–153.11 to 97.11
Early depth severity^c^	–.08	0.10	–0.87	.39	–0.277 to 0.108

a EE: early engagement.

b CE: continued engagement.

c Severity refers to baseline anxiety severity.

#### Frequency (CE)

The overall models were significant for early tasks (*R*^2^=0.014; *P*<.001), early homework (*R*^2^=0.013; *P*<.001), and early depth (*R*^2^=0.015; *P*<.001) predicting CE frequency. However, no significant unique effects for early tasks, early homework, or early depth were found at the Bonferroni-adjusted level, nor were there any interaction effects (Table S2 in Multimedia Appendix 4). Although, in the regression model for early depth predicting CE frequency, there was a trend for a negative relationship between anxiety severity and CE frequency (*r*_2188_=–0.082; *P*<.001), with days between sessions being fewer for those with higher baseline levels of anxiety.

#### Total Homework (CE)

The overall regression models were significant for early homework (*R*^2^=0.215; *P*<.001), early tasks (*R*^2^=0.244; *P*<.001), and early depth (*R*^2^=0.094; *P*=.001) in predicting CE total homework. However, there were no significant unique or interaction effects in any of the models (Table S3 in Multimedia Appendix 4).

## Discussion

### Principal Findings

This study analyzed the relationship between demographic and EE factors, and OE and CE, respectively, and assessed whether such relationships were moderated by baseline anxiety severity in a dMH self-help intervention for adolescent anxiety, BRAVE Self-Help. Importantly, this study examined the utility of conceptualizing engagement, specifically, program use, in multiple ways to better understand engagement in dMH interventions. Overall, the results highlighted that a higher level of EE in the first two sessions was associated with greater CE (intervention-specific factor). This was evident specifically when predicting CE program depth (characters written), but a trend was also observed for CE tasks completed. That is, early depth of program use (characters written in the first two sessions) and early tasks completed in the first two sessions were associated with greater CE in terms of program tasks and depth of responses. In addition, there were bivariate associations between multiple EE and CE variables, suggesting a clear relationship.

Additionally, contrary to expectations, the study failed to demonstrate that anxiety severity impacts engagement either directly or indirectly for most indicators of engagement, although a higher baseline level of anxiety was associated with fewer average days between sessions (frequency). This may suggest that adolescents with more severe anxiety are eager to seek further assistance and see improvements in their anxiety. Although the impact of anxiety severity on engagement was inconsistent in this study, previous research has found that higher anxiety severity is related to poorer program engagement for adolescents in dMH programs [4,16,20,21]. Variations in the way in which engagement has been measured across studies may account for the difference in findings in this study.

Importantly, person-specific demographic factors (age, gender, and location) showed little relevance in predicting program engagement in this study. Previous studies have reported mixed findings with respect to the impact of demographic variables on engagement in dMH programs [4,16,21]. Further research in relation to the relevance of person-specific factors in predicting program engagement should be undertaken once engagement can be measured more holistically.

### EE Appears Important

The intervention-specific finding that EE was generally associated with CE is not surprising, yet an important research finding. Whilst previous research has identified that program engagement is associated with positive clinical outcomes [4,8], this study shows that engaging well early in the program, specifically early program use, is specifically associated with greater CE. Although the effects were small, our findings highlight that there is a need to identify ways to meaningfully engage adolescents in both in-session and homework tasks in the first two sessions of the program, to increase program engagement, and ultimately, the benefits obtained from dMH interventions.

There are few studies that have identified that EE, in terms of use, is important in predicting program engagement, but these studies did not capture the full breadth of engagement (ie, as proposed by Perski et al [15]) and were blended-care or therapist-supported interventions, rather than self-help. In a blended-care CBT intervention for adults with anxiety and depression, where participants had both face-to-face video therapy and digital activities (eg, thought monitoring and mood diaries), participants were around 10 times more likely to withdraw from the intervention if they had not completed the assigned dMH activities after the first session [17]. However, unlike this study, Wu et al [17] defined engagement purely as the number of sessions and web-based activities completed. Another study by Wallert et al [18] demonstrated the importance of early use factors in a therapist-supported dMH program, however, that was limited to early word count and homework tasks only. The study by Wu et al [17] found that higher word count in the first homework tasks was associated with greater adherence (measured in terms of completed homework activities). Interestingly, EE factors included in this study were not associated with completed sessions, but rather, what adolescents did in the program (ie, the amount of in-session and homework activities and character count). This may reflect that participants get what they want or need, without having to complete the entire program (ie, symptom relief or education about anxiety). Alternatively, these findings may suggest that we have not adequately measured all aspects of engagement that relate to treatment outcomes. These results are consistent with findings by both Alberts et al [26] and Yardley et al [14] who found that program engagement may not necessarily predict clinical outcomes. Overall, this study’s findings emphasize the importance of understanding more about EE, its relationship to program completion and outcomes, and the strategies to enhance it.

### Defining Engagement

Contemporary models of engagement highlight the need to consider both user experience and use [14,15]. This study, whilst meaningful in highlighting the importance of EE (program use) in dMH programs for adolescents, did not capture all facets of engagement (eg, user experience), which may explain the small effect sizes found. There is a large variation in the way in which engagement is defined and measured across dMH programs [12], and engagement has not often been measured holistically. This study primarily focused on the use aspects of engagement rather than the experiential or affective elements. The assessment of user experience would allow the opportunity for a more nuanced understanding of engagement, where participants’ own subjective experience of the program is measured [14,15]. For example, this could include self-report questionnaires or interviews on engagement and experience with the program, or examination of the content and quality of activity responses rather than just character count [15], which may allow a richer understanding of the factors predicting OE, reasons for disengagement, and inform the development or refinement of dMH programs to achieve higher levels of engagement.

### Strengths and Limitations

This study holds several strengths. First, the study had a large sample size of adolescents taking part in a real-world effectiveness study. The study further explored novel predictive factors of engagement in dMH programs, filling a significant gap in the literature regarding our understanding of adolescent engagement, particularly adolescent use, in these programs. The results were also analyzed using both a conservative and traditional significance level, adding merit to our findings. Despite its strengths, there were some limitations inherent to the study. First, this study did not consider the predictors of people who fail to take up BRAVE Self-Help, for example, factors at registration, or in the first session, such as participant perceptions of the content being valuable and helpful, time available to complete the program, and beliefs about the need for treatment. These were not the focus of this study; however, they should be the focus of future research, with specific adaptations to collect data of this nature. Furthermore, while this study considered engagement as multifaceted, the components of engagement analyzed in this study were limited to the available data that has been routinely collected within the BRAVE Self-Help platform, and thus, not all components of engagement as suggested in the engagement models of Perski et al [15] and Yardley et al [14] could be examined. Specifically, this study focused on understanding multiple measures of program use, but did not address user experience, which should be analyzed in future research. Further, whilst this study did examine the role of anxiety severity in influencing engagement, it did not examine whether changes in anxiety experienced beyond EE and over the course of treatment, had additional influence on OE. It is possible that as anxiety symptoms reduce, participants would be more or less likely to continue to engage in the program, and this should be examined in future research. Finally, whilst not possible to include all potential psychosocial factors in this study and analysis, engagement may have been influenced by other factors, such as satisfaction with the program, personality, socioeconomic status, and previous exposure to treatment.

### Future Directions

This study highlights the need to better understand how to measure the construct of engagement and investigate the role of EE in supporting OE. It is recognized that facets of engagement may overlap and be interrelated (eg, completed tasks and completed sessions), and therefore, conducting a factor analysis may be useful in identifying whether these variables are discrete, and may assist in the empirical validation of proposed engagement models such as that of Perski et al [15] and Yardley et al [14]. Additionally, analysis of predictors of further potential facets of engagement, such as user experience as proposed by Perski et al [15] (including interest in the program and attention sustained within the program, affect, and satisfaction) will be useful in more broadly understanding the construct of engagement and its relationship to EE, anxiety severity, and outcome. Subsequent studies may also consider the use of digital linguistic tools, which may be useful in providing more information about whether sentiment and affect relate to engagement. Future research should also seek to understand how to enhance EE. This could be achieved through qualitative research with adolescents themselves, to better understand the unique needs of the user, and gain a detailed, nuanced account of how to enhance EE. Adding to our understanding will allow for dMH programs to be refined, tailored, and developed with such findings in mind. Taken together, the application of the findings of this study could improve engagement, and ultimately, potential clinical outcomes for adolescents with anxiety.

### Conclusions

To date, there has been little research investigating the factors influencing engagement in dMH interventions for adolescents. The intervention-specific factor of poor EE, in terms of early program use, was found to be a barrier to CE in this study. Understanding how to better operationalize and measure engagement, along with identifying areas of focus to enhance EE, will help refine adolescent dMH interventions and aid in the design and development of future interventions. By doing so, user engagement can be enhanced and the benefit to the user of the program can be maximized.

## Supplementary material

10.2196/60523 Multimedia Appendix 1 Prescribed tasks and homework tasks across sessions.

10.2196/60523 Multimedia Appendix 2 Correlations between demographic, overall program engagement, continued engagement, and early engagement variables.

10.2196/60523 Multimedia Appendix 3 Moderated regression results of the relationship between demographic variables and OE, and whether these relationships were moderated by anxiety severity.

10.2196/60523 Multimedia Appendix 4 Moderated regression results of the relationship between early engagement and continued engagement, and whether these relationships were moderated by anxiety severity.

## Abbreviations

CAS-8: Children’s Anxiety Scale 8-item
CBT: cognitive behavioral therapy
CE: continued engagement
dMH: digital mental health
EE: early engagement
OE: overall program engagement
