# Supplementary material for: The Role of Early Engagement in a Self-Directed, Digital Mental Health Intervention for Adolescent Anxiety: Moderated Regression Analysis
Source: JMIR Pediatr Parent. 2025 Jun 2;8:e60523. doi: 10.2196/60523 (PMC12148243; doi:10.2196/60523)
Supplement: Multimedia Appendix 1 [file pediatrics-v8-e60523-s001.docx]

| Session | Prescribed Tasks | Homework Tasks^a^ |
| --- | --- | --- |
| 1 | 10 | 0 |
| 2 | 32 | 4 |
| 3 | 17 | 8 |
| 4 | 37 | 16 |
| 5 | 0 | 18 |
| 6 | 4 | 14 |
| 7 | 24 | 8 |
| 8 | 49 | 29 |
| 9 | 51 | 4 |
| 10 | 20 | 13 |
| Total | 244 | 114 |

^a^Homework tasks were prescribed in the previous session, but were not typed into the program until the start of the following session (e.g. Homework for session 1 was prescribed at the end of session 1, but session 1 homework responses were not collected until the start of session 2 after the participant had time to complete the homework).
