# Supplementary material for: The Role of Early Engagement in a Self-Directed, Digital Mental Health Intervention for Adolescent Anxiety: Moderated Regression Analysis
Source: JMIR Pediatr Parent. 2025 Jun 2;8:e60523. doi: 10.2196/60523 (PMC12148243; doi:10.2196/60523)
Supplement: Multimedia Appendix 2 [file pediatrics-v8-e60523-s002.docx]

|  | | 1. | 2. | | 3. | | 4. | | 5. | | 6. | | 7. | | 8. | | 9. | | 10. | | 11. | | 12. | | 13. | | 14. | | 15. | 16. | 17. |
| --- | --- | --- | --- | --- | --- | --- | --- | --- | --- | --- | --- | --- | --- | --- | --- | --- | --- | --- | --- | --- | --- | --- | --- | --- | --- | --- | --- | --- | --- | --- | --- |
| 1. Program Depth (CE) | *r* | 1 | |  | |  | |  | |  | |  | |  | |  | |  | |  | |  | |  | |  | |  |  |  |  |
| 1. Early Tasks | *r* | .178^**^ | | 1 | |  | |  | |  | |  | |  | |  | |  | |  | |  | |  | |  | |  |  |  |  |
| 1. Early Depth | *r* | .278^**^ | | .479^**^ | | 1 | |  | |  | |  | |  | |  | |  | |  | |  | |  | |  | |  |  |  |  |
| 1. Total Homework Tasks (CE) | *r* | .580^**^ | | .493^**^ | | .300^**^ | | 1 | |  | |  | |  | |  | |  | |  | |  | |  | |  | |  |  |  |  |
| 1. Completed Sessions (CE) | *r* | .429^**^ | | -.041 | | -.102^**^ | | -.055 | | 1 | |  | |  | |  | |  | |  | |  | |  | |  | |  |  |  |  |
| 1. Total Tasks (CE) | *r* | .610^**^ | | .555^**^ | | .405^**^ | | .780^**^ | | -.179 | | 1 | |  | |  | |  | |  | |  | |  | |  | |  |  |  |  |
| 1. Average Frequency (CE) | *r* | .101^**^ | | .086^**^ | | .093^**^ | | .301^**^ | | -.120^**^ | | .291^**^ | | 1 | |  | |  | |  | |  | |  | |  | |  |  |  |  |
| 1. Age | *r* | -.033 | | -.053^**^ | | -.001 | | .115 | | -.028 | | .058 | | -.053 | | 1 | |  | |  | |  | |  | |  | |  |  |  |  |
| 1. Gender | *r* | .065^**^ | | .161^**^ | | .194^**^ | | .123 | | -.056 | | .261^**^ | | -.003 | | .084^**^ | | 1 | |  | |  | |  | |  | |  |  |  |  |
| 1. Location | *r* | -.015 | | .000 | | -.032 | | .011 | | .002 | | -.048 | | .027 | | .013 | | -.052^**^ | | 1 | |  | |  | |  | |  |  |  |  |
| 1. Severity | *r* | -.053^**^ | | .001 | | -.036 | | -.078 | | -.011 | | -.045 | | -.082^**^ | | .122^**^ | | -.021 | | -.002 | | 1 | |  | |  | |  |  |  |  |
| 1. Completed Sessions (OE) | *r* | .617^**^ | | -.023 | | -.070^**^ | | -.181 | | .990^**^ | | -.301^**^ | | -.051 | | -.059^**^ | | -.068^**^ | | .005 | | -.032 | | 1 | |  | |  |  |  |  |
| 1. Total Tasks (OE) | *r* | .790^**^ | | .265^**^ | | .091^**^ | | .780^**^ | | .834^**^ | | .971^**^ | | .015 | | -.077^**^ | | .012 | | -.015 | | -.036 | | .875^**^ | | 1 | |  |  |  |  |
| 1. Total Homework (OE) | *r* | .821^**^ | | .306^**^ | | .119^**^ | | .982^**^ | | .717^**^ | | .817^**^ | | .048 | | -.077^**^ | | .015 | | -.007 | | -.038 | | .806^**^ | | .971^**^ | | 1 |  |  |  |
| 1. Early Homework (OE) | *r* | .154^**^ | | .930^**^ | | .420^**^ | | .463^**^ | | -.042 | | .481^**^ | | .079^**^ | | -.035 | | .137^**^ | | .016 | | .007 | | -.029 | | .236^**^ | | .301^**^ | 1 |  |  |
| 1. Program Depth (OE) | *r* | .926^**^ | | .255^**^ | | .474^**^ | | .475^**^ | | .522^**^ | | .596^**^ | | .079^**^ | | -.039 | | .101^**^ | | -.029 | | -.049^**^ | | .650^**^ | | .800^**^ | | .796^**^ | .215^**^ | 1 |  |
| 1. Frequency (OE) | *r* | .048 | | .052^**^ | | .073^**^ | | .295^**^ | | -.110^**^ | | .274^**^ | | .860^**^ | | -.063^**^ | | .005 | | .020 | | -.054^**^ | | -.050^**^ | | -.010 | | .011 | .048^**^ | .039 | 1 |
