# Supplementary material for: The Role of Early Engagement in a Self-Directed, Digital Mental Health Intervention for Adolescent Anxiety: Moderated Regression Analysis
Source: JMIR Pediatr Parent. 2025 Jun 2;8:e60523. doi: 10.2196/60523 (PMC12148243; doi:10.2196/60523)
Supplement: Multimedia Appendix 3 [file pediatrics-v8-e60523-s003.docx]

**Table S1.** Linear Model of Demographic Variables Predicting Completed Sessions (OE) as a Function of Anxiety Severity.

| Demographic  Predictors |  | *B* | *SE B* | *t* | *P* | 95% CI |
| --- | --- | --- | --- | --- | --- | --- |
| Age |  |  |  |  |  |  |
|  | Constant | 4.47 | 1.28 | 3.50 | .001 | 1.97,7.00 |
|  | Age | -.02 | .10 | -.17 | .87 | -.19,.16 |
|  | Severity | .40 | .76 | .52 | .60 | -1.09,1.88 |
|  | Age*Severity | -.04 | .05 | -.71 | .48 | -.143,.067 |
| Gender |  |  |  |  |  |  |
|  | Constant | 4.78 | .34 | 13.90 | <.001 | 4.10,5.45 |
|  | Gender | -.63 | .40 | -1.56 | .12 | -1.41,.16 |
|  | Severity | -.28 | .20 | -1.44 | .15 | -.67,10 |
|  | Gender*Severity | .14 | .23 | .61 | .54 | -.31,.59 |
| Location |  |  |  |  |  |  |
|  | Constant | 5.95 | .46 | 12.95 | <.001 | 5.05,6.85 |
|  | Location | -.99 | .55 | -1.81 | .07 | -2.07,.09 |
|  | Severity | -.87 | .75 | -1.15 | .25 | -2.34,.61 |
|  | Location*Severity | -.32 | .26 | -1.22 | .29 | -.84,.19 |
| *Note.* Severity refers to baseline anxiety severity. | |  |  |  |  |  |

**Table S2.** Linear Model of Demographic Variables Predicting Total Tasks (OE) as a Function of Anxiety Severity.

| Demographic  Predictors |  | *B* | *SE B* | *t* | *P* | 95% CI |
| --- | --- | --- | --- | --- | --- | --- |
| Age |  |  |  |  |  |  |
|  | Constant | 81.83 | 20.01 | 4.09 | <.001 | 42.59,121.08 |
|  | Age | -.60 | 1.43 | -.41 | .68 | -3.38,2.20 |
|  | Severity | 6.37 | 11.90 | 5.54 | .60 | -17.00.29.69 |
|  | Age*Severity | -.62 | .84 | -.74 | .46 | -2.27,1.03 |
| Gender |  |  |  |  |  |  |
|  | Constant | 95.57 | 6.28 | 15.38 | <.001 | 84.25,108.89 |
|  | Gender | -6.69 | 7.40 | -.90 | .38 | -21.20,7.82 |
|  | Severity | -6.25 | 3.60 | -1.74 | .08 | -13.30,.80 |
|  | Gender *Severity | 6.63 | 4.25 | 1.56 | .12 | -1.71,14.97 |
| Location |  |  |  |  |  |  |
|  | Constant | 98.57 | 6.85 | 14.19 | <.001 | 84.94,112.20 |
|  | Location | -13.38 | 8.31 | -1.61 | .11 | -29.68,2.92 |
|  | Severity | -8.01 | 11.39 | -.70 | .48 | -30.34,14.32 |
|  | Location*Severity | -3.25 | 3.97 | -.82 | .41 | -11.04,4.54 |

*Note.* Severity refers to baseline anxiety severity.

**Table S3.** Linear Model of Demographic Variables Predicting Program Depth (OE) as a Function of Anxiety Severity.

| Demographic  Predictors |  | *B* | *SE B* | *t* | *P* | 95% CI |
| --- | --- | --- | --- | --- | --- | --- |
| Age |  |  |  |  |  |  |
|  | Constant | 12.42 | .58 | 21.50 | <.001 | 11.29,13.55 |
|  | Age | .38 | .29 | 1.28 | .12 | -.19,.95 |
|  | Severity | -.60 | .33 | -1.80 | .07 | -1.25,.05 |
|  | Age*Severity | -.24 | .18 | -1.31 | .19 | -.59,.12 |
| Gender |  |  |  |  |  |  |
|  | Constant | 11.06 | 1.06 | 10.42 | <.001 | 8.98,13.15 |
|  | Gender | 1.67 | 1.25 | 1.33 | .18 | -.79,4.12 |
|  | Severity | -.77 | .61 | -1.27 | .20 | -1.97,.42 |
|  | Gender*Severity | .38 | .72 | .52 | .60 | -1.03,1.79 |
| Location |  |  |  |  |  |  |
|  | Constant | 12.26 | 1.19 | 10.32 | <.001 | 9.93,14.60 |
|  | Location | -.42 | 1.42 | -.29 | .77 | -3.21,2.37 |
|  | Severity | .67 | 1.95 | .35 | .73 | -3.15,4.49 |
|  | Location*Severity | -.23 | .68 | -.34 | .73 | -1.56,1.10 |

*Note.* Severity refers to baseline anxiety severity.

**Table S4.** Linear Model of Demographic Variables Predicting Frequency (OE) as a Function of Anxiety Severity.

| Demographic  Predictors |  | *B* | *SE B* | *t* | *P* | 95% CI |
| --- | --- | --- | --- | --- | --- | --- |
| Age |  |  |  |  |  |  |
|  | Constant | 12.25 | 4.15 | 2.95 | .003 | 4.10,20.39 |
|  | Age | -.42 | .30 | -1.41 | .16 | -.997,.163 |
|  | Severity | -2.22 | 2.47 | -.90 | .37 | -7.06,2.62 |
|  | Age*Severity | .01 | .17 | .56 | .57 | -.24,.44 |
| Gender |  |  |  |  |  |  |
|  | Constant | 7.45 | 1.12 | 6.65 | <.001 | 5.25,9.64 |
|  | Gender | -1.18 | 1.30 | -.90 | .36 | -3.75,1.38 |
|  | Severity | -1.51 | .64 | -2.36 | .02 | -2.76,-.26 |
|  | Gender*Severity | .75 | .75 | .10 | .32 | -.72,2.21 |
| Location |  |  |  |  |  |  |
|  | Constant | 5.33 | 1.22 | 4.34 | <.001 | 2.93,7.74 |
|  | Location | 1.76 | 1.48 | 1.19 | .23 | -1.14,4.66 |
|  | Severity | 3.84 | 2.05 | 1.87 | .06 | -.18,7.86 |
|  | Location*Severity | -.27 | .70 | -.39 | .70 | -1.64,1.10 |

*Note.* Severity refers to baseline anxiety severity.

**Table S5.** Linear Model of Demographic Variables Predicting Total Homework (OE) as a Function of Anxiety Severity

| Demographic  Predictors |  | *B* | *SE B* | *t* | *P* | 95% CI |
| --- | --- | --- | --- | --- | --- | --- |
| Age |  |  |  |  |  |  |
|  | Constant | 67.01 | 2.47 | 27.12 | <.001 | 62.16,71.85 |
|  | Age | 1.12 | 1.25 | .89 | .37 | -1.34,3.58 |
|  | Severity | -1.04 | 1.43 | -.73 | .47 | -3.84,1.77 |
|  | Age*Severity | -1.15 | .77 | -1.50 | .13 | -2.66,.36 |
| Gender |  |  |  |  |  |  |
|  | Constant | 55.70 | 4.08 | 13.64 | <.001 | 47.66,63.68 |
|  | Gender | -2.65 | 4.77 | -.56 | .58 | -12.01,6.70 |
|  | Severity | -3.40 | 2.32 | -1.72 | .09 | -8.55,.56 |
|  | Gender*Severity | 2.16 | 2.72 | .79 | .44 | -3.19,7.50 |
| Location |  |  |  |  |  |  |
|  | Constant | 69.81 | 5.08 | 13.74 | <.001 | 59.84,79.77 |
|  | Location | -7.79 | 6.09 | -1.28 | .12 | -19.71,4.13 |
|  | Severity | .12 | 8.33 | .01 | .99 | -16.22,16.48 |
|  | Location*Severity | -1.53 | 2.91 | -.53 | .60 | -7.23,4.17 |

*Note.* Severity refers to baseline anxiety severity.
