# Supplementary material for: The Role of Early Engagement in a Self-Directed, Digital Mental Health Intervention for Adolescent Anxiety: Moderated Regression Analysis
Source: JMIR Pediatr Parent. 2025 Jun 2;8:e60523. doi: 10.2196/60523 (PMC12148243; doi:10.2196/60523)
Supplement: Multimedia Appendix 4 [file pediatrics-v8-e60523-s004.docx]

**Table S1.** Linear Model of EE Variables Predicting Overall Completed Sessions (CE) as a Function of Anxiety Severity

| EE Predictors |  | *B* | *SE B* | *t* | *P* | 95% CI |
| --- | --- | --- | --- | --- | --- | --- |
| Early Tasks |  |  |  |  |  |  |
|  | Constant | 3.99 | .99 | 4.04 | <.001 | 2.05,5.954 |
|  | Early Tasks | .04 | .03 | 1.26 | .21 | -.02,.09 |
|  | Severity | .92 | .56 | 1.64 | .10 | -.18,2.02 |
|  | Early Tasks * Severity | -.03 | .02 | -1.77 | .08 | -.06,.00 |
| Early Homework |  |  |  |  |  |  |
|  | Constant | 4.23 | .78 | 5.44 | <.001 | 2.71,5.76 |
|  | Early Homework | .05 | .03 | 1.30 | .20 | -.02,.11 |
|  | Severity | .73 | .44 | 1.65 | .10 | -.14,1.60 |
|  | Early Homework * Severity | -.04 | .02 | -1.83 | .07 | -.075,.003 |
| Early Depth |  |  |  |  |  |  |
|  | Constant | 5.74 | .40 | 14.40 | <.001 | 4.50,6.52 |
|  | Early Depth | -.00 | .00 | -1.44 | .15 | -.00,.00 |
|  | Severity | -.12 | .23 | -.54 | .59 | -.57,.33 |
|  | Early Depth * Severity | .00 | .00 | .24 | .81 | -.00,.00 |

*Note.* Severity refers to baseline anxiety severity.

**Table S2.** Linear Model of EE Variables Predicting Frequency (CE) as a Function of Anxiety Severity

| EE  Predictors |  | *B* | *SE B* | *t* | *P* | 95% CI |
| --- | --- | --- | --- | --- | --- | --- |
| Early Tasks |  |  |  |  |  |  |
|  | Constant | 3.81 | 3.60 | 1.06 | .29 | -3.24,10.88 |
|  | Early Tasks | .12 | .10 | 1.19 | .23 | -.08,.33 |
|  | Severity | -1.23 | 2.02 | -.61 | .54 | -5.20,2.74 |
|  | Early Tasks * Severity | -.01 | .06 | -.19 | .85 | -.12,.10 |
| Early Homework |  |  |  |  |  |  |
|  | Constant | 4.86 | 2.82 | 1.72 | .09 | -.67,10.40 |
|  | Early Homework | .15 | .13 | 1.17 | .24 | -.10,.40 |
|  | Severity | -1.28 | 1.60 | -.80 | .42 | -4.41,1.85 |
|  | Early Homework * Baseline anxiety severity | -.02 | .07 | -.22 | .83 | -.15,.12 |
| Early Depth |  |  |  |  |  |  |
|  | Constant | 7.79 | 1.46 | 5.32 | <.001 | 4.92, 10.66 |
|  | Early Depth | .00 | .00 | 1.33 | .89 | -.00,.00 |
|  | Severity | -2.29 | .83 | -2.75 | .006 | -3.93,-.66 |
|  | Early Depth * Severity | .00 | .00 | 1.04 | .30 | -.00,.00 |

*Note.* Severity refers to baseline anxiety severity.

**Table S3.** Linear Model of EE Variables Predicting Total Homework (CE) as a Function of Anxiety Severity

| EE  Predictors |  | *B* | *SE B* | *t* | *P* | 95% CI |
| --- | --- | --- | --- | --- | --- | --- |
| Early Tasks |  |  |  |  |  |  |
|  | Constant | 45.95 | 29.03 | 1.58 | .16 | -11.38,103.27 |
|  | Early Tasks | 1.13 | .79 | 1.42 | .16 | -.44,2.70 |
|  | Severity | -9.19 | 16.51 | -.56 | .58 | -41.78, 23.40 |
|  | Early Tasks * Severity | .26 | .46 | .56 | .58 | -.65,1.16 |
| Early Homework |  |  |  |  |  |  |
|  | Constant | 48.10 | 26.76 | 1.80 | .07 | -4.73, 100.94 |
|  | Early Homework | 1.66 | 1.14 | 1.46 | .15 | -.59,3.91 |
|  | Severity | -3.60 | 14.90 | -.24 | .81 | -33.01,25.82 |
|  | Early Homework * Baseline anxiety severity | .16 | .64 | .25 | .80 | -1.10.1.43 |
| Early Depth |  |  |  |  |  |  |
|  | Constant | 80.29 | 13.62 | 5.90 | <.001 | 53.40,107.18 |
|  | Early Depth | .02 | .02 | .76 | .45 | -.03,.06 |
|  | Severity | -5.95 | 7.80 | -.75 | .46 | -21.73,9.82 |
|  | Early Depth * Severity | .01 | .01 | .43 | .66 | -.02,.03 |

*Note.* Severity refers to baseline anxiety severity.
